# 

**Published:** 1878-04-20

**Authors:** 


### CONTENTS.

### ORIGINAL AND SELECTED ARTICLES.

Early Rupture of Membranes: by W S Posey. M.D., of Sulphur Bluffs. Texas................ 98
The Atlanta Medico-Chirurgical Association.. 99
Corrosive Sublimate in Dysentery; by. Chas. H. Hall. M.D., of Macon. Ga....................101
The Use of Cold and Heat in Fevers; by Frank Alport, M.D.. Sycamore. Ill.....................103
The Preventive Treatment of Puerperal Fever; by S. E. Robinson, M.D., of West Union, Iowa..........................................106
Bromide of Potassium in the uncontrollable Vomiting of Pregnancy; by Samuel C. Busey, M.D , Washington, D. C..............107
Ovariotomy..................................................110
A Case of Post-Partum Hemorrhage treated by Hyatt's Method......................................111

### ABSTRACTS AND GLEANINGS.

Iodoform, 112; Tannin in a Case of Very Intractable Vomiting during Pregnancy, 112; The Iodide of Ethyl in the Treatment of Asthma, 113; Medical Aphorisms. 113; Polypus of the Uterus, 115; Carbolate of Soda in the Treatment of Nervous Affections of the Respiratory Passages, 115; Vaccination with Horse-Lymph, 116; Treatment of Cracked Nipples 116; Glycerine in the Treatment of Internal Hemorrhoids. 116; Toxic Properties of Dynamite, 116; Menstruation and Ovulation. 117; Treatment of Epilepsy, 117; The Danger of Inflammable Volatile Liquids, 117; Malt Extract as an Emulsifier, 118; Belladonna in Dysentery, 118; Ovulation without Menstruation, 118; Nitrite of Amyl as an Antiperiodic, 118; Grindelia Robusta in Whooping Cough, 119; A Prize of $80.000, 119; Summer Complaint. 119; Borax and Nitrate of Potassium in Sudden Hoarseness, 120; Stibiodermic Treatment in Acute Articular Rheumatism, 120; Purulent Ophthalmia of Infants, 120; Feeding per Rectum, 120; Strychnia in Bronchitis, 121; Belladonna in Collapse, 121: The Treatment of Burns by Bicarbonate of Soda, 121; The Removal of Moles, 121; M. Guerin's Treatment of Carbuncle, 121; Ergot in Diabetes Insipidus, 121.

### PRACTICAL NOTES AND FORMULÆ.

Laceration of the Perineum, 122; Burns, 122; Pruritus Vulvæ, 123: Arsenic in Skin Diseases, 124; Guiacum in Quinsy, 124; Hemorrhoids, 124; Yerba Reuma, 124; Tannin as a Medical Agent, 124; In Menorrhagia, 124: Night Sweats, 124; Colliquative Diarrhœa, 124.

### SCIENTIFIC ITEMS.

The Electric Light in Paris, 125; Disgust Philosophically Considered, 125; Telegraphing without Wires, 125; Persistence of the Retinal Image, 125.

### EDITORIAL AND MISCELLANEOUS.

Medical Literature, etc., 126; Does Cinchoquinine Embody all the Properties of the Cinchona Bark? 126; Proceedings of the Louisiana Medical Association, 127; Measles, 128; Book Notices, 128.

